# Node Deployment of Marine Monitoring Networks: A Multiobjective Optimization Scheme

**DOI:** 10.3390/s20164480

**Published:** 2020-08-11

**Authors:** Jian-Li Duan, Bin Lin, Lin X. Cai, Yu-Xiang Liu, Yuan Wu

**Affiliations:** 1 School of Information and Communication Engineering, Dalian Maritime University, Dalian 116026, China; duanjianli@qut.edu.cn (J.-L.D.); bybyby285@sina.com (Y.-X.L.); 2 School of Science, Qingdao University of Technology, Qingdao 266033, China; 3 Department of Electrical and Computer Engineering, Illinois Institute of Technology, Chicago, IL 60616, USA; lincai@iit.edu; 4 School of Science, Macao University, Macao 999078, China; yuanwu@um.edu.mo

**Keywords:** marine monitoring networks, multiobjective optimization, network deployment, ant colony algorithm, gurobi

## Abstract

The increasing demands for real-time marine monitoring call for the wide deployment of Marine Monitoring Networks (MMNs). The low-rate underwater communications over a long distance, long propagation delay of underwater acoustic channel, and high deployment costs of marine sensors in a large-scale three-dimensional space bring great challenges in the network deployment and management of MMN. In this paper, we first propose a multitier, hierarchical network architecture of MMN with the support of edge computing (HMMN-EC) to enable efficient monitoring services in a harsh marine environment, taking into consideration the salient features of marine communications. Specifically, HMMN-EC is composed of three subnetworks, i.e., underwater acoustic subnetwork, the sea-surface wireless subnetwork, and the air wireless subnetwork, with a diversity of network nodes with different capabilities. We then jointly investigate the deployment diverse network nodes with various constraints in different subnetworks of HMMN-EC. To this end, we formulate a Multiobjective Optimization (MO) problem to minimize the network deployment cost while achieving the maximal network lifetime, subject to the limited energy of different marine nodes and the complex deployment environment. To solve the formulated problem, we present an Ant-Colony-based Efficient Topology Optimization (AC-ETO) algorithm to find the optimal locations of nodes in different subnetworks of MMN in a large-scale deployment. The time complexity of the proposed algorithm is also analyzed. Finally, extensive simulations are carried out to validate the superior performance of the proposed algorithm compared with some existing solutions.

## 1. Introduction

With the deepening of humans’ understanding of the ocean, as well as the rapid development of science and technology, great attention has been paid to the ocean because of its huge economic potential and strategic importance. The increasing demand for the exploitation and utilization of marine resources calls for the wide deployment of marine monitoring networks. For example, a large number of drilling platforms have been built at sea to extract oil from the sea [1]. However, the exploitation of offshore oil resources also brings pollution risk to the marine environment. In 2010, the Gulf of Mexico oil spill accident led to serious harm to the marine ecosystem [2]. In such case, an underwater monitoring network is helpful to detect the oil spill and report the detection results in a timely manner. Another application is real-time monitoring for marine ranching, which is heavily dependent on the quality of marine environment to foster the marine fishery resources [3]. Thus motivated, a real-time Marine Monitoring Network (MMN) has become an important research topic for both academia and industry. Node deployment is one of the fundamental tasks for MMN, and also is an attractive research topic. During the past decade, many technologies and systems related to marine monitoring have been developed, such as a buoy for marine monitoring [4,5], a prediction model of battery life [6], and a data acquisition and transmission system [7], which build a foundation for the implementation of the real-time MMN. According to the requirements of marine monitoring, various types sensors are deployed to monitor and measure different physical and chemical parameters such as water temperature, pressure, water direction and speed, salinity, turbidity, pH, oxygen density, and chlorophyll levels [4], and then the acquired data are transmitted back to the data center on land by relay nodes. The data acquired from the seabed far away from the coast needs to be relayed back to the data center through multi-layer relays. Compared with terrestrial monitoring networks, deployment of an MMN is more costly and complex due to the harsh marine environment in three-dimensional space.

Most existing works of the deployment of marine monitoring sensor networks in the literature proposed different algorithms to improve the network coverage. In [8], a distributed node deployment algorithm was proposed to utilize the mobility of the anchor nodes to maximize the coverage of 3D underwater wireless sensor networks in dynamic ocean environments. In [9,10,11,12], different algorithms were proposed to deploy sensors and surface gateways in a underwater sensor network. These works mainly focus on the deployment of underwater acoustic networks (UANs). To enable marine monitoring service, it is also critical to forward the data of the UAN towards the Base Station (BS) which is usually deployed in the shoreline of ocean. For efficient data communications over the large area of ocean, a multitier network that incorporates both underwater acoustic communications, radio communications above the water, and aerial relay over the air, is highly desirable. To the best of our knowledge, no existing work on the deployment of marine monitoring network study the deployment of an integrated multitier hierarchical network architecture, which includes underwater acoustic subnetwork, sea-surface wireless subnetwork, and air wireless subnetwork.

**Figure 1:**
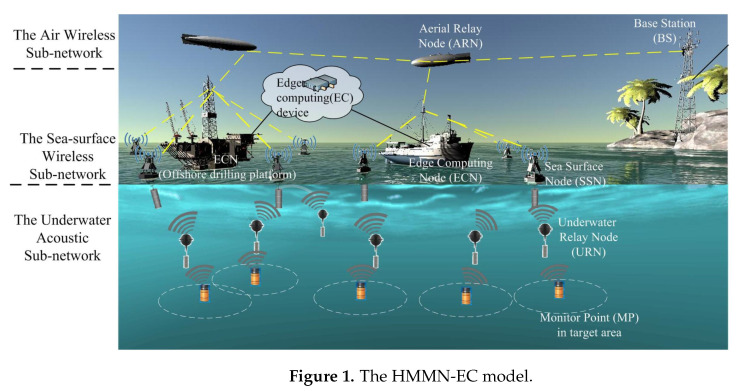
The HMMN-EC model.

In this paper, we first propose a three-tier hierarchical network architecture of MMN with support of edge computing (HMMN-EC), as shown in Figure 1, for an integrated sea–air–ground monitoring system. In the HMMN-EC, the underwater acoustic subnetwork consists of a number of battery-powered sensors with limited energy [13]—Autonomous Underwater Vehicles (AUVs) [14] and buoys [15] with acoustic receiving devices and RF transmitting devices; the sea-surface wireless subnetwork consists of unmanned ships which carry RF communication devices; and the air wireless subnetwork [16,17] consists of Aerial Relay Nodes (ARNs), such as Unmanned Air Vehicles (UAVs) with RF communication equipment. Under the proposed network architecture, the node deployment problem is further investigated to achieve the minimum network cost while ensuring the maximum network lifetime. Specifically, a Multiobjective Optimization (MO) problem is formulated to minimize the costs and maximize the network lifetime by deploying different types of nodes in different tiers of the network, considering the energy and capacity constraints of each node. The formulated optimization problem can be solved by Gorubi. As Gurobi does not work well when the network is scaled up, we propose a swarm-intelligent-based optimization approach to find the near-optimal solution of the formulated optimization problem. The main contributions are summarized as follows.

(1) A novel integrated multitier hierarchical network architecture of MMN with support of edge computing is proposed, namely, HMMN-EC which integrates the UAN, the sea-surface wireless network with edge computing, and the air wireless network.

(2) Based on the hierarchical network architecture, a multiobjective optimization framework is formulated to minimize the network deployment cost while maximizing the network lifetime by determining the deployment locations of network nodes, including ARNs, Edge Computing Nodes (ECNs), Sea-Surface Nodes (SSNs), and Underwater Relay Nodes (URNs), and the data transmission links between network nodes, subject to various constraints of the network topology, network connectivity, and the battery capacity.

(3) An Ant-Colony-based Efficient Topology Optimization (AC-ETO) algorithm is presented to solve the formulated MO problem in various network scenarios of different numbers of nodes.

(4) Extensive simulations are conducted to validate the performance of the proposed algorithm. The results show that the proposed algorithm approaches the optimal solution and outperforms some existing solutions.

The rest of this paper is organized as follows. In Section 2, we review the related works about the deployment of MMN. Section 3 describes the network model and presents the problem formulation. In Section 4, an AC-ETO algorithm is proposed. Section 5 shows the numerical analysis, followed by concluding remarks in Section 6.

## 2. Related Works

Most researches of deployment of MMN focus on node deployment of UAN networks, i.e., sensors and/or surface gateways. Ibrahim S., Cui J., and Ammar R. formulated the optimal gateway deployment problem as an Integer Linear Programming (ILP) problem in [10]. They propose an algorithm to deploy multiple surface-level gateways in [12], and use a greedy algorithm to select gateway positions among candidate locations. In these works, they mainly study sensor deployment under 2D space. In [18], Song X. and Gong Y. et al. proposed a 3D node deployment algorithm for underwater sensor networks. The proposed algorithm can achieve a large coverage area with the minimal number of nodes. In [19], Jiang P., Wang X., and Jiang L. proposed a depth adjustment algorithm based on connected tree (CTDA), in which the sink node is used as the first root node of a connected tree, and the whole network is organized as a forest that comprises many connected subtrees. To maximize the network coverage, coverage overlaps between the parent node and the child node are reduced within each subtree. In addition, in [9,20,21], Han G. and Pompili D. et al. presented 2D and 3D communication architectures, and review deployment algorithms and strategies for UANs from different perspectives. It is found that most existing works focus on the node deployment of underwater subnetwork nodes, and few works jointly consider the network deployment of underwater network and network above the water surface.

With the popularity of Swarm Intelligence (SI), a number of researches propose to use SI- and SI-based algorithms (SIAs) to tackle the optimization problems in node deployment of traditional wireless sensor networks and communication networks. Ant Colonies Optimization (ACO) is one of the well-known representative SIAs, where complex collective behavior emerges from the behavior of ants. ACO is effective for solving Non-deterministic Polynomial (NP) hard discrete optimization problems, and has been successfully applied to a number of scientific and engineering problems, including grid-based deployment for wireless sensor networks [22,23,24]. ACO is also applied to the topology optimization [25,26] and routing algorithm [27,28] for wireless networks. An ACO algorithm coupled with a local search heuristic is proposed in [29] to deploy a WSN under a certain reliability constraint at the minimum deployment cost. However, algorithms for traditional WSN or communication networks cannot be directly applied for MMNs due to the different characteristics of the deployment environment of ocean in three-dimensional space. To this end, we are motivated to apply ACO for the MMN deployment under three-tier architecture, and formulate a multiobjective optimization problem characterizing the 3D marine environment.

## 3. Network Model and Problem Formulation

### 3.1. Network Model

The hierarchical network model of HMMN-EC is composed of three subnetworks: (1) the underwater acoustic subnetwork, (2) the sea-surface wireless subnetwork, and (3) the air wireless subnetwork, as shown in Figure 1.

In an underwater acoustic subnetwork, multiple sensors deployed at representative Monitoring Points (MPs) are deployed to monitor the target areas. The MP then transmits the monitoring data to a SSN, typically via one or multiple URNs, when communication distance is beyond the transmission range of sensors at MPs. URN is a buoyancy-driven device which can hover and select a specified position to acquire and transmit data over acoustic communication channels [30,31,32]. The location of URN should be carefully decided as it is dependent on the influence of ocean flow and undercurrent. SSU is equipped with a wireless communication radio installed on the buoy, an acoustic–electric conversion device, and an underwater acoustic receiver located under the sea surface. After receiving the underwater acoustic signals from URNs, it converts them into radio signals and then transmits radio signals to an ECN or other SSN within its communication distance.

The sea-surface wireless subnetwork is comprised of multiple ECNs, which are responsible for receiving radio signals from the underwater acoustic subnetwork, processing the data in an edge device, and sending the processed data to the air wireless subnetwork. Generally, unmanned ships with communication equipment and small edge servers are used as ECNs.

The air wireless subnetwork further relays the received data from the the sea-surface wireless subnetwork to the BS. This subnetwork consists of multiple ARNs that transmit the received data to the BS over one or multiple hops through other ARNs. Finally, the BS transmits the data from the HMMN-EC network to the data center through the terrestrial wireless networks.

In the HMMN-EC, the BS is the destination node; ARNs, ECNs, SSNs, and URNs are the intermediate nodes; and MPs are source nodes. All nodes are organized hierarchically within the communication radius of nodes, and an efficient tree architecture will finally be formed to achieve an effective communication.

In summary, various nodes involved in the HMMN-EC have a certain communication radius, by using either radio or acoustic communications; and each node only communicate with other nodes of the same subnetwork or nodes of the upper subnetwork within their communication distance. In addition, the ARN and ECN nodes may have sufficient power supply with no stringent capacity limitation, but battery-powered SSN and URN nodes are typically of small sizes and thus are subject to certain battery constraints, which should be taken into consideration for network deployment.

### 3.2. Energy Model

In the HMMN-EC, ARNs and ECNs usually have sufficient energy supply with no stringent capacity limitation; while battery-powered underwater nodes, i.e., SSNs, URNs, and MPs, are typically of small sizes with limited battery capacity. Thus, to provision quality marine monitoring services of the HMMN-EC, it is of critical importance to improve the operation time of the underwater nodes.

Generally, the states of the battery of a node include sending, receiving, idle, and sleeping. It is reported in [33] that the communication module consumes the most energy, i.e., around 80% of the total energy consumption. Energy consumption during idle and sleep modes is only related to time. In our system model, energy consumption per unit of time during idle and sleep mode is regarded as a constant. Here, an URN communicates to other URNs or SSNs over underwater acoustic channels.

The transmission and receiving energy consumption of node *i* over a communication channel, i.e., either an acoustic channel [34] or a radio channel [35], are denoted as $E_{tr}^{i}$ and $E_{re}^{i}$, respectively, which are given by

(1) $$E_{tr}^{i}\left(l_{ij},d_{ij}\right)=\left\{\begin{array}{c} E_{0}d_{ij}^{k}10^{d_{ij}\frac{\alpha (f)}{10}}l_{ij},\;\;\mathrm{acoustic}\;\mathrm{channel},\\ E_{\mathrm{elec}}l_{ij}+\varepsilon_{amp}l_{ij}d_{ij}^{2},\;\mathrm{radio}\;\mathrm{channel}, \end{array}\right.$$

and

(2) $$E_{re}^{i}\left(l_{ti}\right)=\left\{\begin{array}{c} E_{r}l_{ti},\;\;\;\;\mathrm{acoustic}\;\mathrm{channel},\\ E_{\mathrm{elec}}l_{ti},\;\;\;\mathrm{radio}\;\mathrm{channel}, \end{array}\right.$$

where $E_{\mathrm{elec}}$ is the energy consumption of the transmitter circuit; $\varepsilon_{amp}$ is the energy consumption of power amplifier; $E_{0}$ is the energy consumption of transmitting one bit of data with a certain communication radius; $E_{r}$ is the energy consumption of receiving one bit of data; *k* is the energy diffusion factor; $l_{ij}$ is the size of the data packet from node i to node j in bits; and $d_{ij}$ is the transmission distance from node i to node j. α(*f*) is the Doppler frequency-absorption coefficient of signal frequency *f*, which is given by Throp [34],

$$\alpha \left(f\right)=\frac{0.11f^{2}}{1+f^{2}}+\frac{44f^{2}}{4100+f^{2}}+2.75\times 10^{-4}f^{2}+0.003.$$

Thus, the communication energy consumption of node *i* is

(3) $${\mathrm{Ec}}_{i}=\sum_{j\in V}E_{tr}^{i}\left(l_{ij},d_{ij}\right)e_{ij}+\sum_{t\in V}E_{re}^{i}\left(l_{ti}\right)e_{ti},$$

where $e_{ij}=1$ indicates that node i can directly communicate with node j and vice versa. The total energy consumption of node i is

(4) $$E_{i}={\mathrm{Ec}}_{i}+E_{\mathrm{idle}}^{i}+E_{\mathrm{sleep}}^{i},$$

where $E_{\mathrm{idle}}^{i}$ and $E_{\mathrm{sleep}}^{i}$ are the energy consumption of node i during idle and sleep mode, respectively.

### 3.3. Problem Formulation

We model the HMMN-EC as a directed graph $\vec{G}=\left(V,\vec{E}\right)$, where V represents the set of nodes, i.e., BSs, ARNs, ECNs, SSNs, URNs, and MPs, and $\vec{E}$ represents directed edges between two nodes that are within the communication radius. To differentiate nodes, the subsets of BSs, ARNs, ECNs, SSNs, URNs, and MPs are denoted as $V_{\mathrm{BS}},V_{\mathrm{ARN}},V_{\mathrm{ECN}},V_{\mathrm{SSN}}$, $V_{\mathrm{URN}}$, and $V_{\mathrm{MP}}$. Thus, the whole set of nodes $V=V_{\mathrm{BS}}\cup V_{\mathrm{ARN}}\cup V_{\mathrm{ECN}}\cup V_{\mathrm{SSN}}\cup V_{\mathrm{URN}}\cup V_{\mathrm{MP}}$. The edge $e_{ij}\in \vec{E}$ is a binary variable, $e_{ij}=1$ indicates there exists a direct communication link from node i to node j, and vice versa. $|V_{x}|$ represents the number of nodes in set $V_{x}$. The main notations used in the paper are listed in Table 1.

As the marine monitoring devices—especially the battery-powered underwater monitoring devices—are expensive, it is desirable to reduce the total number of devices for deployment to minimize the total deployment cost. In the meantime, it is hard if not impossible to replace batteries of underwater nodes, and thus, it is important to maximize the operation time of network nodes. In this paper, we will formulate MO problem under the HMMN-EC architecture. The primary objectives are to minimize the total deployment cost while maximizing the network lifetime subjected to the limited node communication radius and battery capacities. Here, the network lifetime is defined as the time until the first node runs out of energy [36].

#### 3.3.1. Minimization of the Total Network Deployment Cost

The first objective is to minimize $C_{net}$, the total deployment cost of the network, i.e., the sum deployment cost of ARNs, ECNs, SSNs, and URNs. The MPs are predeployed based on the marine areas of interest, while other types of nodes are deployed to collect and forward the information from MPs to the Internet servers. Denote the unit deployment cost of ARN, ECN, SSU, and URN as $C^{\mathrm{ARN}},C^{\mathrm{ECN}},C^{\mathrm{SSN}},$ and $C^{\mathrm{URN}}$. Thus, we have

(5) $$C_{net}=C^{\mathrm{URN}}\sum_{m\in V_{\mathrm{URN}}}a_{m}+C^{\mathrm{SSN}}\sum_{n\in V_{\mathrm{SSN}}}b_{n}+C^{\mathrm{ECN}}\sum_{l\in V_{\mathrm{ECN}}}h_{l}+C^{\mathrm{ARN}}\sum_{t\in V_{\mathrm{ARN}}}z_{t},$$

where $a_{m}$, $b_{n}$, $h_{l}$, $z_{t}$ are binary variables of Candidate Locations (CLs) of URNs, SSNs, ECNs, and ARNs, respectively. The value of 1 indicates that the CL is selected to place a corresponding node, and vice versa.

#### 3.3.2. Maximization of the Network Lifetime

Besides the network deployment cost, it is also critical to ensure that the HMMN-EC provisions quality marine monitoring services as long as possible. The network lifetime is defined as the operation time of the network until the first battery-powered node exhausts the energy supply and become out of service. Given the initial battery of a battery-powered node *i*, ${\mathrm{EI}}_{i}$, and the energy consumption of node *i*, $E_{i}$ per unit time, the lifetime of node *i* is then given by

(6) $$T_{i}=\frac{{\mathrm{EI}}_{i}}{E_{i}},\forall i\in V_{\mathrm{URN}}\cup V_{\mathrm{SSN}}.$$

Therefore, the network lifetime $T_{net}$ is defined as

(7) $$T_{net}=\min_{\forall i\in V_{\mathrm{URN}}\cup V_{\mathrm{SSN}}}T_{i}=\min_{\forall i\in V_{\mathrm{URN}}\cup V_{\mathrm{SSN}}}\frac{{\mathrm{EI}}_{i}}{E_{i}}.$$

Notice that $E_{i}$ is dependent on the communication distance and the communication data volume shown as (1)–(4). Thus, the network lifetime is determined by ${\mathrm{Ec}}_{i}$ of the first energy-exhausted node i. Accordingly, to maximize the network lifetime, it is equivalent to minimize the energy consumption of the first energy-exhausted node. According to (3) and (4), the energy consumption of the first energy-exhausted node is given by

(8) $$E_{\max}=\max_{i\in V_{\mathrm{URN}}\cup V_{\mathrm{SSN}}}\left(\sum_{j\in V}E_{tr}^{i}\left(l_{ij},d_{ij}\right)e_{ij}+\sum_{t\in V}E_{re}^{i}\left(l_{ti}\right)e_{ti},+E_{\mathrm{idle}}^{i}+E_{\mathrm{sleep}}^{i}\right).$$

Without the loss of generality, the initial energy of node (EI) is regarded as 100% in the following formulation.

Thus, the MO problem is formulated as follows:

(9) $$P1:\;\mathrm{minimize}\;C_{net}+\omega E_{max},$$

(10) $$s.t.\sum_{j\in V_{\mathrm{URN}}}e_{ij}\geq K,\forall i\in V_{\mathrm{MP}},$$

(11) $$\;\sum_{i\in V_{\mathrm{MP}}\cup V_{URN}}e_{im}\geq a_{m},\forall m\in V_{\mathrm{URN}},m\neq i,$$

(12) $$\;\sum_{j\in V_{\mathrm{URN}}\cup V_{\mathrm{SSN}}}e_{mj}=a_{m},\forall m\in V_{\mathrm{URN}},m\neq j,$$

(13) $$\;\sum_{j\in V_{\mathrm{URN}}\cup V_{\mathrm{SSN}}}e_{jn}\geq b_{n},\forall n\in V_{\mathrm{SSN}},n\neq j,$$

(14) $$\;\sum_{q\in V_{\mathrm{SSN}}\cup V_{ECN}}e_{nq}=b_{n},\forall n\in V_{\mathrm{SSN}},n\neq q,$$

(15) $$\;\sum_{q\in V_{\mathrm{SSN}}\cup V_{\mathrm{ECN}}}e_{ql}\geq h_{l},\forall l\in V_{\mathrm{ECN}},l\neq q,$$

(16) $$\;\sum_{u\in V_{\mathrm{ECN}}\cup V_{ARN}}e_{lu}=h_{l},\forall l\in V_{\mathrm{ECN}},l\neq u,$$

(17) $$\;\sum_{u\in V_{\mathrm{ECN}}\cup V_{\mathrm{ARN}}}e_{ut}\geq z_{t},\forall t\in V_{\mathrm{ARN}},t\neq u,$$

(18) $$\;\sum_{s\in V_{\mathrm{ARN}}\cup V_{BS}}e_{ts}=z_{t},\forall t\in V_{\mathrm{ARN}},t\neq s,$$

(19) $$\;\sum_{i\in V_{\mathrm{URN}}}f_{ij}+\sum_{k\in V_{\mathrm{MP}}}g_{k}e_{kj}=\sum_{l\in V_{\mathrm{URN}}}f_{jl}+\sum_{m\in V_{\mathrm{SSU}}}f_{jm},\forall j\in V_{URN},i\neq j,j\neq l,$$

(20) $$\;\sum_{i\in V_{\mathrm{SSN}}}f_{ij}+\sum_{k\in V_{\mathrm{URN}}}f_{kj}=\sum_{l\in V_{\mathrm{SSN}}}f_{jl}+\sum_{m\in V_{\mathrm{ECN}}}f_{jm},\forall j\in V_{\mathrm{SSN}},i\neq j,j\neq l.$$

Equation (9) is the weighted sum of the two main objectives, where the network cost $C_{net}$ and the energy consumption of the first exhausted node $E_{max}$ are defined in (5) and (8), respectively, and ω is the weight to strike a balance of the two objectives.

Constraint (10) specifies that each MP must be covered by at least K URNs. Different monitoring tasks have different requirements of K. Equation (11) indicates that if CL *j* is selected to deploy a URN, then $a_{j}=1$, and there exists at least one link of receiving data, e.g., between a MP or URN node *i* and *j*. Equation (12) indicates that when an URN *j* is deployed, there exists one link of forwarding data, e.g., between node *j* to another URN or SSN nodes *l*. Similarly, SSNs, ECNs, and ARNs are subject to the constraints (13)–(18), respectively. Equations (13) and (14) specify that if a SSU *n* is deployed, i.e., $b_{n}=1$, the SSU *n* has one link of forwarding data and at least one link of receiving data. Similarly, Equations (15) and (16) specify that if CL *l* is selected to deploy an ECN, i.e., $h_{l}=1$, there exists one link of forwarding data and at least one link of receiving data. Equations (17) and (18) specify that if CL *t* is selected to deploy an ARN, i.e., $z_{t}=1$, there exists one link of forwarding data and at least one link of receiving data. Equations (19) and (20) specify the flow constraint that the output data should be the same as the input data.

## 4. Ant-Colony-Based Efficient Topology Optimization (AC-ETO)

### 4.1. Algorithm Description

As the formulated optimization problem is an integer linear programming problem, which is known to be NP-hard [37]. The ant colony is widely used to solve various NP-hard problems. especially [38]. Thus, in this section, we propose an Ant-Colony-based efficient topology optimization algorithm, namely, AC-ETO, to solve the proposed problem in P1.

In a traditional ACO algorithm, ants choose the next city through a probabilistic rule, and then iteratively construct the best path [39]. The probability for an ant to move from city *i* to city *j* is

(21) $$\begin{array}{c} \;\;p_{ij}=\frac{{\left(\tau_{ij}\right)}^{\alpha }\eta_{ij}^{\beta }}{\sum_{j\in V_{\mathrm{allowed}}}{\left(\tau_{is}+\tau_{is}^{\prime }\right)}^{\alpha }\eta_{is}^{\beta }}, \end{array}$$

where $\tau_{ij}$ is the amount of pheromone deposited for a transition from city *i* to *j*; α is a parameter to control the influence of $\tau_{ij}$; $\eta_{ij}$ is a heuristic factor for the transition from city *i* to *j* and is typically inversely proportional to the distance between cities *i* and *j*, i.e., $\eta_{ij}=1/d_{ij}$; β is a parameter to control the influence of $\eta_{ij}$; and $V_{\mathrm{allowed}}$ is the feasible neighborhood of an ant in city *i*.

In AC-ETO, we select data forwarding paths in the hierarchical HMMN-EC step-by-step. In each step, a probabilistic transition rule is applied to select a deployment location. For example, at node *i*, the probability that the deployment location *j* (*j* = 1,2,...|$V_{\mathrm{FCL}}^{i}$|) is selected is given by

(22) $$\begin{array}{c} \;\;p_{ij}=\frac{(\tau_{ij}^{\prime }+{\tau_{ij}^{\prime \prime })}^{\alpha }{\eta_{ij}^{\prime }}^{\beta }}{\sum_{j\in V_{\mathrm{FCL}}^{i}}{\left(\tau_{ij}^{\prime }+\tau_{ij}^{\prime \prime }\right)}^{\alpha }{\eta_{ij}^{\prime }}^{\beta }}, \end{array}$$

where $\tau_{ij}^{\prime }$ given in Formula (23) is the global pheromone trail value between node *i* and node *j*; $\tau_{ij}^{\prime \prime }$ given in Formula (24) is the local value between the two nodes *i* and *j*; $\eta_{ij}^{\prime }$ is the heuristic value of adding node *j* to the connected cover currently being built by the ant, which is defined as Formula (25); and α and β are parameters that control the influence of the pheromone trail values and heuristic information on $p_{ij}$, respectively. $V_{\mathrm{FCL}}^{i}$ is the Feasible Candidate Location (FCL) set of node *i*, and FCL is defined as CLs within the communication range of a node.

(23) $$\begin{array}{c} \;\;\tau_{ij}^{\prime }=\left(1-\rho_{1}\right)\tau_{ij}^{\prime }+\Delta \tau_{ij}^{\prime }, \end{array}$$

(24) $$\begin{array}{c} \;\;\tau_{ij}^{\prime \prime }=\left(1-\rho_{2}\right)\tau_{ij}^{\prime \prime }+\Delta \tau_{ij}^{\prime \prime }, \end{array}$$

(25) $$\begin{array}{c} \;\;\eta_{ij}^{\prime }=\left\{\begin{array}{cc} \frac{1}{E_{tr}^{i}} & i\in V_{URN}\cup V_{SSN}\\ 1 & otherwise \end{array}\right. \end{array}$$

where $E_{tr}^{i}$ is given by Formula (1). $\Delta \tau_{ij}^{\prime }$ and $\Delta \tau_{ij}^{\prime \prime }$ are defined as follows:

(26) $$\begin{array}{c} \;\;\Delta \tau_{ij}^{\prime }=\left\{\begin{array}{cc} \frac{Q-C^{j}}{C_{bestnet}} & e_{ij}=1\\ 0 & otherwise \end{array}\right. \end{array}$$

(27) $$\begin{array}{c} \;\;\Delta \tau_{ij}^{\prime \prime }=\left\{\begin{array}{cc} \frac{Q-C^{j}}{C_{path}} & e_{ij}=1\\ 0 & otherwise \end{array}\right. \end{array}$$

where $C^{j}$ is the cost of node *j*, $C_{bestnet}$ is the current minimum network cost, and $C_{path}$ is the cost of the path. *Q* is a constant greater than 1, and $\rho_{1}$ and $\rho_{2}$ are the rates of global and local pheromone evaporation.

The process of AC-ETO is divided into two phases. The first phase is the initialization phase, which reduces the search space so that only FCLs are selected and stored by certain nodes. The second phase is the planning phase, where the optimization process is iteratively performed to construct the network topology and remove the redundant edges until the desired result is reached.

**Figure 2:**
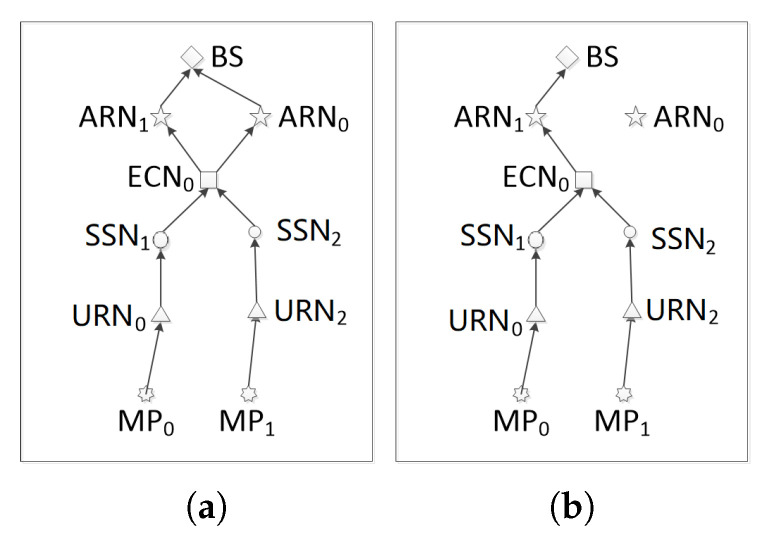
The topology of the network with 2 MPs; (**a**) Before removing; (**b**) After removing.

We first use a small-scale network with 2 MPs as an example to describe the algorithm. In the initialization phase, the distances between nodes are calculated based on the location of nodes. Each node then constructs a FCL table which includes all nodes in its communication coverage. For instance, the FCL table of MP0 includes URN0 and URN1. In the planning phase, a number of iterations are involved. In each iteration, a number of ants are placed on each MP to construct paths to the BS by using the probabilistic rule, the local pheromone, and the global pheromone defined in (22)–(24). An ant placed on MP0 moves to the next node, e.g., URN0 in the FCL table according to the pheromones and the transition probability until it arrives at the BS. After the ant reaches the BS, the BS informs all nodes along the path to update the local pheromone and selects the best path from multiple ants, e.g., MP0-URN1-SSN0-ECN0-ARN1-BS0 and MP1-URN2-SSN2-ECN0-ARN0-BS0 in the 2-MP example, as shown in Figure 2a. Notice that the two paths are independently found by ants and there may be multiple links between two nodes, e.g., ECN0-ARN1-BS0 and ECN0-ARN0-BS0. In such case, the two links are compared and the path with higher energy consumption, e.g., ECN0-ARN0-BS0, is removed to obtain a tree with $C_{net}$ + $\omega E_{\max}$ = 68.417, as shown in Figure 2b. The iteration repeats until no better tree with a smaller $C_{net}$ + $\omega E_{\max}$ can be found.

The pseudo code of the proposed AC-ETO is elaborated in Algorithm 1.

**Algorithm 1** The pseudo code of AC-ETO algorithm
**Input:**
$V_{\mathrm{MP}},V_{\mathrm{URN}}V_{\mathrm{SSN}},V_{\mathrm{ECN}},V_{\mathrm{ARN}},V_{\mathrm{BS}}$
1: **Phase I: Initialization**2: Input positions of nodes and other parameters;3: N←|V|;4: $D_{N\times N}\leftarrow the\;\;distance\;\;between\;\;nodes$;5: **for**
i=1 to *N*
**do**6:  $T_{Feabile}^{i}\leftarrow FCLtable\_Bld\left(i\right)$;7: **end for**8: **Phase II: Multi-objective planning**9: ${M\_Global}_{N\times N}\leftarrow \left\{1\right\}$;10: $V_{start}\leftarrow V_{\mathrm{MP}}$, $M\leftarrow |V_{\mathrm{MP}}|$;11: **repeat**12:  **Step 1-Network construction**13:  **for**
i=1 to *M*
**do**14:   Take a MP j randomly from $V_{\mathrm{start}}$;15:   Place m ants on j; \\ m is a constant integer16:   **for** each ant r=1 to *m*
**do**17:    ${M\_Local}_{N\times N}\leftarrow \left\{1\right\}$;18:    Put j into node_list of r: $V_{list}^{r}\leftarrow V_{list}^{r}\cup \left\{j\right\}$;19:    k←j;20:    ${M\_prob}_{N\times N}\leftarrow \left\{0\right\}$;21:    **while** ($l\notin V_{\mathrm{BS}}$) **do**22:     Choose and move to next node k from $T_{Feabile}^{k}$;23:     $V_{list}^{r}\leftarrow V_{list}^{r}\cup \left\{k\right\}$;24:    **end while**25:    Calculate the cost of $V_{list}^{r}$;26:    Calculate the energy consumption of each node t:${EC}_{t}$;27:   **end for**28:   Choose the best path for MP j from $\left\{V_{list}^{1},\ldots ,V_{list}^{m}\right\}\to {list}_{j}$;29:   The Cost of ${list}_{j}\to C_{j}$;30:   Calculate the energy consumption of each node t:${EC}_{t}$;31:   Update ${M\_Local}_{N\times N}$;32:  **end for** 
\\
**Step 2-Redundant edge removal**33:  Remove redundant edges from the initial constructed network;34:  Update ${M\_Global}_{N\times N}$;35: **until** iterative number > ψ)36: **Output:** the optimal solution, total cost, and energy consumption;

### 4.2. Computational Complexity Analysis

In this subsection, we analyze the computational complexity of the proposed (AC-ETO) algorithm.

Phase I: Initialization (Lines 1–7): In the initialization phase, the locations of a number of network nodes, including BS, MPs, CLs of ARNs, ECNs, SSNs and URNs, are imported. Accordingly, the network parameters such as communication distance, initial energy, and transmit and receive power are set. The complexity of initialization is O(N), where *N* is the network size. Then, the distance matrix between neighboring nodes are calculated first, and the complexity is $O(N^{2})$. According to the distance matrix, each node maintains an FCL table that includes the list of nodes that it can directly communicate with. For example, an URN list a set of other URNs and/or SSNs in its communication coverage. The worst-case complexity is O((N−1)(N−M)). Therefore, the complexity of Phase I is $O(2N^{2}-MN-N+M)$.

Phase II: Topology planning (Lines 8–34): Based on the FCL tables calculated in phase I, an AC-basedoptimal method is used to find the placement of the minimum cost and the energy consumption. The algorithm is iteratively performed for network construction and redundant edge removal until a desirable result is reached. In Lines 9–10, the matrix of global pheromone is initialized to 1 by the memset function with a complexity of O(N), and the subset $V_{\mathrm{MP}}$, which has *M* elements, is set as the set of starting points $V_{\mathrm{start}}$ and the complexity is O(M), where M is the number of MPs. In Lines 11–34, the iterative optimization process is executed, where Lines 12–31 are for Step 1—Network construction, and Lines 32–33 are for Step 2—Redundant edge removal. In Step 1, an ant colony is placed on a MP which is randomly selected from $V_{\mathrm{MP}}$, and then moves to construct paths towards the BS. By comparing the values of (9) for each feasible path, the best one—the one with the minimum value of (9)—is selected and stored. When *M* ant colonies complete path construction, the sequences of *M* best paths are selected to construct a network. Step 1 yields the worst case complexity $O(MN^{2}+MN+M^{2})$. In Step 2 (Lines 32–33), the result of Step 1 is modified by removing some redundant edges according to the characteristics of the tree structure constraints, and the complexity of this step is $O(N^{2}+M^{2}+M)$. Figure 2 shows an example of Step 2. Figure 2a shows the constructed network topology after step 1. It can be seen that the structure is not a tree topology as the out degree of $ECN_{0}$ is 2. In Figure 2b, two redundant edges are removed from the network to form a tree topology. Thus, the complexity of Lines 12–33 is as follows.

$$\begin{array}{c} O\left(MN^{2}+MN+M^{2}\right)+O\left(N^{2}+M^{2}+M\right)=O\left(MN^{2}+N^{2}+MN+2M^{2}+M\right). \end{array}$$

Accordingly, if *N* is sufficiently large, the complexity of Phase II is approximately $O(\psi MN^{2}+\psi MN+\psi M^{2}+\psi M)$, where ψ is the maximum number of iterations.

Therefore, the overall computation complexity of the algorithm is $O(2N^{2}-MN-N+M)$+$O\left(\psi MN^{2}+\psi MN+\psi M^{2}+\psi M\right)\approx O\left(\psi MN^{2}+\psi MN+\psi M^{2}\right)$. The AC-ETO is efficient and achieves a polynomial time complexity.

## 5. Simulations and Discussion

**Figure 3:**
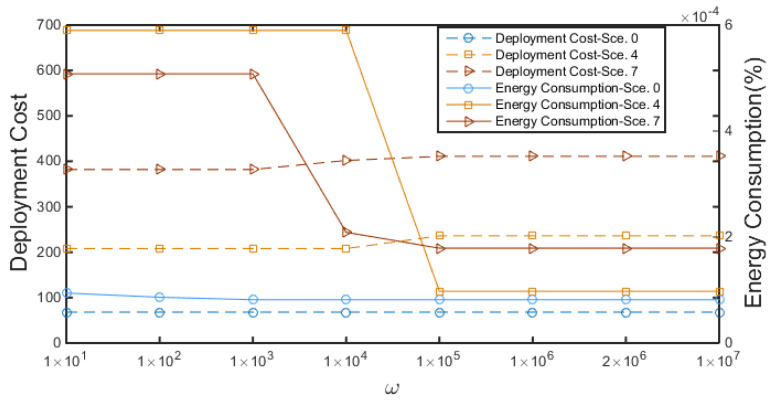
The impacts of ω: DC stands for Deployment Cost, and EC stands for Energy Consumption. Sce. is the abbreviation of Scenario.

In this section, we validate the performance of the proposed algorithm and compare it with benchmark algorithms in different network scenarios. MPs are pre-defined carefully in the monitoring sea area according to sea state conditions and needs. Specifically, we first validate the performance in a small-scale network. Then, we show the performance of MO problem solved by Gurobi [40] in small-scale networks and compare it with our proposed algorithm. A greedy algorithm is further presented and compared with the proposed AC-ETO algorithm. We set up multiple experiments of eleven network scenarios of different scales, as shown in Table 2. The main parameters used in the experiments are listed in Table 3. Without loss of generality, the generic cost unit (gcu) and the generic time unit (gtu) are defined to simplify the evaluation of deployment costs and network lifetime in the case studies. The impacts of ω on the results are tested in different scenarios under various network scales, as shown in Figure 3. It can be seen that the deployment cost shows little variance, but the energy consumption may decrease significantly when ω increases. In other experiments, the value of ω is set to 2 × 10${}^{6}$.

### 5.1. Performance Validation in Small-Scale and Medium-Scale Networks

**Figure 4:**
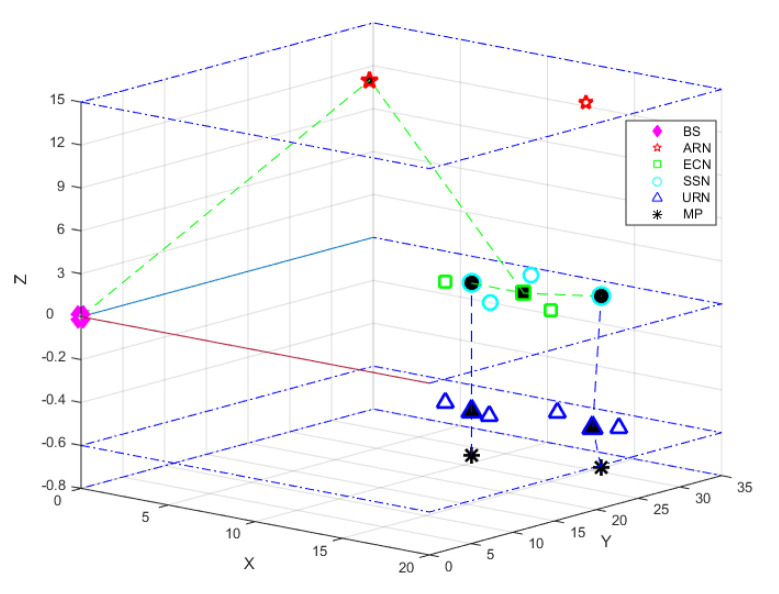
The optimal solution of Scenario ($C_{net}=68$ gcu, $E_{max}=8.18\times 10^{-5}\%$).

We first evaluate the network performance of small-scale network scenarios with a small number of nodes, i.e., Scenario 0 and Scenario 1. Let us take Scenario 0 as an example. The results obtained by Gurobi in Figure 4 show the deployment solution of Scenario 0. The deployment cost of this solution is 68, which is the minimum cost in Scenario 0. Similarly, the energy consumption is 8.18 ×$10^{-5}$, which is also the lowest one among all deployment plans. In this simple case, the optimization solution obtained by Gurobi is optimal, compared with the results obtained from the exhaustive search.

**Figure 5:**
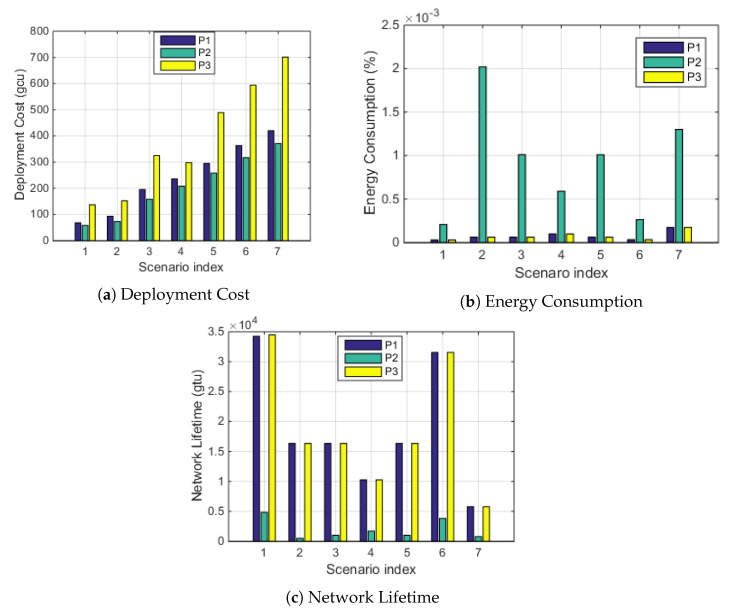
Comparison of P1, P2, and P3 in terms of the deployment cost, energy consumption, and network lifetime.

We then compare the solutions of the MO problem (P1) with each of the subproblems of MO, i.e., to minimize the cost (P2) or to minimize the energy consumption (P3) under different scenarios from scenario 1 to 7. The results are compared in Figure 5. As shown in Figure 5a, the deployment cost of P1 is slightly greater than that of P2, but smaller than that of P3. Figure 5b shows that the energy consumption of P1 is similar to that of P3, but much smaller than that of P2. Correspondingly, the network lifetime of P1 is much greater than that of P2, while smaller-than or equal-to that of P3, as shown in Figure 5c. Thus, using P1, a longer network lifetime can be achieved with a lower deployment cost. Again, Gurobi can achieve the optimal solution.

**Figure 6:**
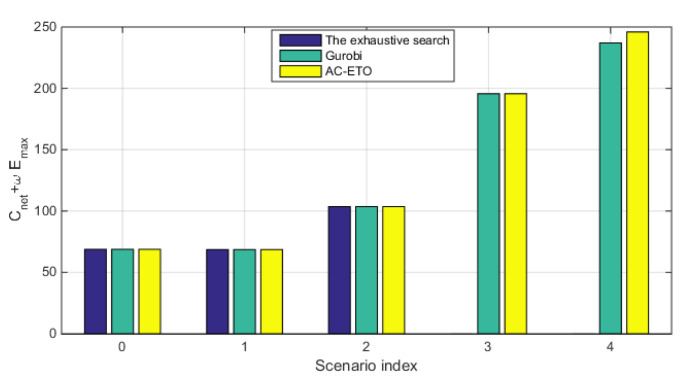
Comparison of the exhaustive search, Gurobi and the AC-ETO in terms of the optimization objective.

Based on the above process, a series of small-scale network simulation are carried out to verify the performance of the algorithm and compare it with the exhaustive search and Gurobi. As shown in Figure 6, the results of the proposed algorithm in Scenarios 0–2 approaches that of the exhaustive search and Gurobi. For instance, the optimal solution of the AC-ETO in Scenario 0 is 68.818, which equals to that of the exhaustive search. Thus, the solutions of the algorithm in small-scale networks are close to optimal. As the scale of the network increase, it is difficult to obtain the optimal solutions by the exhaustive search. We further compare the solutions of the algorithm with that of the Gurobi in Scenarios 3–4. Figure 6 shows that the results of the algorithm is very close to that of gurobi. Therefore, the optimization solution of the AC-ETO are close to optimal in small-scale and medium-scale networks.

### 5.2. Performance Analysis of Gurobi and AC-ETO in Different Network Scenarios

**Figure 7:**
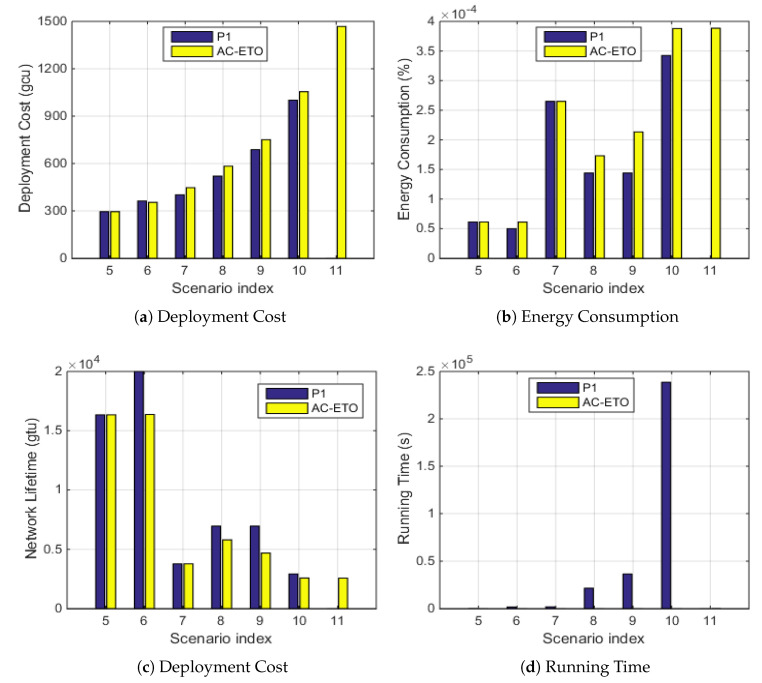
Comparison of Gurobi and AC-ETO in terms of deployment cost, energy consumption, network lifetime, and time complexity under various network scenarios.

We further study the performance of the proposed algorithm AC-ETO under different network scales and compare the results with the solutions of P1 obtained by Gurobi. As shown in Figure 7a, the deployment cost obtained by Gurobi is slightly smaller than that by AC-ETO in Scenarios 5 to 10. Figure 7b,d compare the energy consumption and network lifetime performance obtained by Gurobi and by AC-ETO under different scenarios. Similarly, it can be observed that Gurobi slightly outperform AC-ETO in all these metrics. In Figure 7c, when the network is scaled up with a larger number of nodes, the time complexity of Gurobi increases drastically, while the running time of AC-ETO does not vary much. As an example, in Scenario 9, the energy consumption by AC-ETO is 0.69E-4% greater than that by Gurobi, and the deployment cost by AC-ETO is 9.16% higher than that by Gurobi, but the time complexity of Gurobi is 1000 times higher than that of AC-ETO. As the network size increases, Gurobi cannot obtain the results in the last scenario—i.e., scenario 11—within the set time limit of 300,000 s, yet the proposed AC-ETO algorithm obtains the results efficiently. Thus, the AC-ETO is efficient in dealing with those large-scale network scenarios at the cost of a slight reduction of the optimality.

### 5.3. Performance Comparison of AC-ETO and a Greedy Algorithm

We further compare the performance of the proposed AC-ETO algorithm with a greedy algorithm shown in Algorithm A1. Initially, the feasible table—i.e., FCL Table of each node—is established to store the CLs within the communication coverage. All CLs in the table are grouped by type in increasing order of the communication distance. To set up a path, each node along the path is selected by checking CLs in the FCL Table of the prior node in an order, until the BS is reached. The network construction completes when all paths are found for any MP.

**Figure 8:**
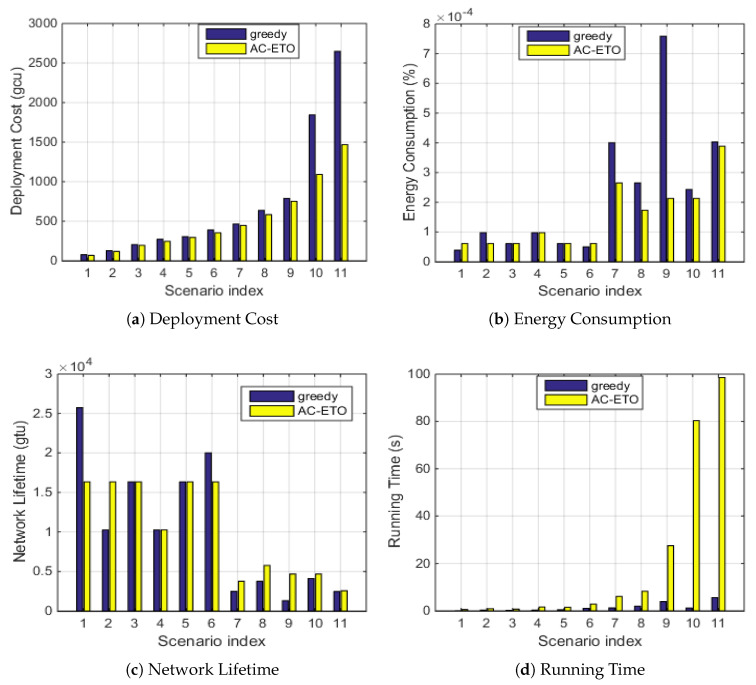
Comparison of AC-ETO and a greedy algorithm in terms of the average deployment cost and energy consumption of twenty execution times under various scenarios.

As shown in Figure 8a, the deployment cost of AC-ETO is lower than that of the greedy algorithm. In Figure 8b, it is found that the energy consumption of the greedy algorithm in different scenarios is higher than that of AC-ETO. Accordingly, the network lifetime of AC-ETO is longer than that of the greedy algorithm in Figure 8c. In the greedy algorithm, it is favorable to select a path with the minimum cost and, accordingly, the minimum energy consumption, which does not guarantee the cost and energy consumption of the overall network. While AC-ETO guides ants to find the optimal (approximate optimal) solution through two pheromones. Figure 8d shows that running time increases with the network size. For instance, in Scenario 7, the deployment cost obtained by AC-ETO is 447 gcu while the deployment cost obtained by greedy algorithm is 841 gcu, and the energy consumption obtained by AC-ETO is 5% less than that obtained by greedy algorithm. Thus, AC-ETO achieves better performance than that of the greedy algorithm in different scenarios, at the cost of increased but affordable time complexity.

Based on the above analysis, it is observed that the AC-ETO algorithm outperforms the greedy algorithm and approaches the optimal solutions in different scenarios which may be difficult for Gurobi to solve.

## 6. Conclusions

In this paper, we have presented a multitier hierarchical network architecture with the support of edge computing that includes the underwater acoustic subnetwork, the sea-surface wireless subnetwork, and the air wireless subnetwork. Based on the network architecture, we have formulated an MO problem to minimize the total network deployment cost and maximize the network lifetime. To solve the MO problem, we have proposed an efficient algorithm, namely, AC-ETO, and analyzed its time complexity. The proposed algorithm approaches the optimal solutions under different network scales with polynomial time. We will jointly study the network deployment of static ocean sensors and the trajectory design of mobile ocean vehicles in our future work.

## Figures and Tables

**Table 1:** Table of Notations.

Symbols	Definition
$g_{i}$	The amount of data of ${\mathrm{MP}}_{i}$(per unit time).
$R_{\mathrm{MP}}$	The perceived radius of MPs.
$D^{\mathrm{ARN}}$	The communication distance of ARNs.
$D^{\mathrm{ECN}}$	The communication distance of ECNs.
$D^{\mathrm{SSN}}$	The communication distance of SSNs.
$D^{\mathrm{URN}}$	The communication distance of URNs.
$C^{\mathrm{ARN}}$	The cost of ARNs.
$C^{\mathrm{ECN}}$	The cost of ECNs.
$C^{\mathrm{SSN}}$	The cost of SSNs.
$C^{\mathrm{URN}}$	The cost of URNs.
$E_{\mathrm{elec}}$	Energy consumption for sending and receiving data per bit.
$P_{\mathrm{UR}}$	The reception power of URNs.
$P_{\mathrm{UT}}$	The transmission power of URNs.
${\mathrm{EI}}_{i}$	The initial energy of node *i*.
${\mathrm{Ec}}_{i}$	The energy consumption of node *i* per unit time.
*K*	K-coverage: each MP must be covered by K URNs.
$\vec{E}={\left\{e_{ij}\right\}}_{\left|V|\times |V\right|},\forall i,j\in V$	The matrix of edge variables, where $e_{ij}\in \left\{0,1\right\}$ is a binary variable and $e_{ij}=1$ denotes node i can directly communicate with node j; and vice versa.
$A={\left\{a_{m}\right\}}_{1\times |V_{\mathrm{URN}}|},\forall m\in V_{\mathrm{URN}}$	The location incidence vector of nodes, where $a_{m}\in \left\{0,1\right\}$ is a binary variable and $a_{m}$ = 1 denotes that the candidate location m is selected to deploy a URN; and vice versa.
$B={\left\{b_{n}\right\}}_{1\times |V_{\mathrm{SSN}}|},\forall n\in V_{\mathrm{SSN}}$	The location incidence vector of nodes, where $b_{n}\in \left\{0,1\right\}$ is a binary variable and $b_{n}$ = 1 denotes that the candidate location n is selected to deploy a SSN; and vice versa.
$H={\left\{h_{l}\right\}}_{1\times |V_{\mathrm{ECN}}|},\forall l\in V_{\mathrm{ECN}}$	The location incidence vector of nodes, where $h_{l}\in \left\{0,1\right\}$ is a binary variable and $h_{l}$ = 1 denotes that the candidate location l is selected to deploy an ECN; and vice versa.
$Z={\left\{z_{t}\right\}}_{1\times |V_{\mathrm{ARN}}|},\forall t\in V_{\mathrm{ARN}}$	The location incidence vector of nodes, where $z_{t}\in \left\{0,1\right\}$ is a binary variable and $z_{t}=1$ denotes that the candidate location *t* is selected to deploy an ARN; and vice versa.
$f_{\mathrm{ij}},\forall i,j\in V$	The data flow from node *i* to node *j*.

**Table 2:** The setting of simulated scenarios. CLs—Candidate Locations.

Scenario Index	Number of					
	BS	MP	CLs of ARN	CLs of ECN	CLs of SSN	CLs of URN
0	1	2	2	3	4	6
1	1	2	1	2	10	15
2	1	4	1	2	20	25
3	1	6	2	8	20	30
4	1	8	2	8	25	40
5	1	10	3	5	35	50
6	1	12	3	10	40	60
7	1	15	3	9	50	70
8	1	20	4	12	80	100
9	1	25	5	16	100	120
10	1	35	7	25	125	150
11	1	50	7	25	170	200

**Table 3:** Parameter Setting.

Parameter	Value
${\mathrm{EI}}_{\mathrm{SSN}},{\mathrm{EI}}_{\mathrm{URN}}$	2, 3 (J)
$E_{r},E_{o}$	10, 50 (nJ)
$E_{\mathrm{elec}}$	50 (nJ)
$D^{\mathrm{URN}},D^{\mathrm{SSN}},D^{\mathrm{ECN}},D^{\mathrm{ARN}}$	5, 10, 25, 30 (km)
$R_{\mathrm{MP}}$	0.2 (km)
$C^{\mathrm{URN}},C^{\mathrm{SSN}},C^{\mathrm{ECN}},C^{\mathrm{ARN}}$	15, 10, 9, 9 (gcu)
$\epsilon_{amp}$	0.84
ω	2 × 10${}^{6}$

## Abbreviations

   The following abbreviations are used in this manuscript:

ARN	Aerial Relay Node
AUV	Autonomous Underwater Vehicle
BS	Base Station
CL	Candidate Location
ECN	Edge Computing Node
FCL	Feasible Candidate Location
MO	Multi-objectives Optimization
MMN	Marine Monitoring Network
RF	Radio Frequency
SSN	Sea-surface Node
UAN	Underwater Acoustic Network
UAV	Unmanned Air Vehicle
URN	Underwater Relay Node

## Appendix A

**Algorithm A1** A Greedy Algorithm
**Require:**$V_{\mathrm{MP}},V_{\mathrm{URN}},V_{\mathrm{SSN}},V_{\mathrm{ECN}},V_{\mathrm{ARN}},V_{\mathrm{BS}}$;
Initialization: Input positions of nodes and other parameters;
**for** each i∈V **do**
Build the feasible table FCL table for *i*;
**end for**
$M\leftarrow |V_{MP}|$
**for** i=1 to *M* **do**
Find a feasible node *l* from FCL table of $MP_{i}$;
**while** ($l\notin V_{\mathrm{BS}}$) **do**
Find a feasible node k from FCL table of *l*;
l←k;
path_list of i←k;
**end while**
**end for**
**for** each path **do**
$V_{\mathrm{net}}\leftarrow path$;
**end for**
Calculate the total cost $C_{\mathrm{net}}$ and the energy consumption $E_{M}\mathrm{ax}$;
**Ensure:**$V_{\mathrm{net}},C_{\mathrm{net}},E_{\mathrm{Max}}$;
